# Effectiveness and safety of self-managed oral anticoagulant therapy compared with direct oral anticoagulants in patients with atrial fibrillation

**DOI:** 10.1038/s41598-018-33531-7

**Published:** 2018-10-25

**Authors:** Erik Lerkevang Grove, Flemming Skjøth, Peter Brønnum Nielsen, Thomas Decker Christensen, Torben Bjerregaard Larsen

**Affiliations:** 1 Department of Cardiology, Aarhus University Hospital, Aarhus, Denmark; 2 Department of Clinical Medicine, Faculty of Health, Aarhus University, Aarhus, Denmark; 3 Aalborg Thrombosis Research Unit, Department of Clinical Medicine, Faculty of Health, Aalborg University, Aalborg, Denmark; 4 Unit of Clinical Biostatistics, Aalborg University Hospital, Aalborg, Denmark; 5 Department of Cardiothoracic and Vascular Surgery, Aarhus University Hospital, Aarhus, Denmark; 6 Department of Cardiology, Aalborg University Hospital, Aalborg, Denmark

## Abstract

We compared the effectiveness and safety of direct oral anticoagulants (DOAC) vs patient self-managed warfarin therapy (PSM) in patients with atrial fibrillation. We linked prospectively registered data from university hospital clinics to nationwide Danish health registries. Primary effectiveness and safety outcomes were ischaemic stroke (incl. systemic embolism) and major bleeding. All-cause mortality and all-cause stroke were secondary outcomes. An inverse probability of treatment propensity-weighted approach was applied to adjust for potential confounding. The study cohorts included 534 patients treated with PSM and 2,671 patients treated with DOAC. Weighted rates of ischaemic stroke were 0.46 and 1.30 percent per year with PSM vs DOAC, hazard ratio (HR) 0.27 (95% confidence interval 0.11–0.68) with 2.5 years follow-up. Rates of major bleeding were 2.32 and 2.13 percent per year (HR 1.06 [0.69–1.63]). All-cause mortality was not statistically different (HR 0.67 [0.39–1.17]), whereas the incidence of all-cause stroke was significantly lower among patients treated with PSM with rates of 0.61 vs 1.45 percent per year (HR 0.36 [0.16–0.78]). In patients with atrial fibrillation, self-managed oral anticoagulant treatment was associated with a significantly lower risk of all-cause and ischaemic stroke compared to treatment with DOAC, whereas no significant differences were observed for major bleeding and mortality.

## Introduction

Atrial fibrillation (AF) increases the risk of stroke by a factor of 4–5 and accounts for almost 15% of all ischaemic strokes^1^. Several studies have demonstrated that the risk of stroke is reduced by oral anticoagulant therapy with vitamin K antagonists (VKA), such as warfarin, or with direct oral anticoagulants (DOAC), also known as non–VKA oral anticoagulants (dabigatran, rivaroxaban, apixaban, and edoxaban)^2–6^. Accordingly, oral anticoagulant treatment, with either VKA or DOAC, remains the optimal approach to reduce the risk of stroke in patients with AF. International guidelines for the management of AF recommend initiation of DOAC over VKA in patients who are eligible for both^7,8^. Furthermore, switching from VKA to DOAC is recommended when the quality of anticoagulation control with warfarin is suboptimal^7^.

Patients on VKA are regularly monitored, and their doses adjusted to ensure that the International Normalised Ratio (INR) scores remain in the therapeutic range. However, this is often challenging due to variation in individual responses and the narrow therapeutic window of warfarin. Point-of-care devices, allowing self-testing of INR with a drop of whole blood, facilitate optimised management by offering frequent INR measurements and reduce the need for attending anticoagulation clinics. Patient self-management (PSM) is a model empowering trained patients to monitor and adjust their treatment in home settings. This strategy has proved effective and convenient compared to conventional treatment with VKA^9,10^. We have recently reported better effectiveness and similar safety in patients with mechanical heart valves^11^ and patients on long-term anticoagulant treatment after recurrent venous thromboembolism (VTE)^12^. Although AF is the most frequent indication for OAC, studies comparing high-quality warfarin treatment with DOAC is lacking. We aimed to compare the effectiveness and safety of self-managed OAC and treatment with DOAC for stroke prevention in a real-life setting of patients with AF.

## Materials and Methods

This was an observational cohort study investigating warfarin-treated AF patients assigned to PSM compared with matched DOAC-treated patients using Danish registry data.

### Patients and data collection

We studied AF patients assigned to PSM at Center for Self-Managed Oral Anticoagulation, Department of CardioThoracic and Vascular Surgery, Aarhus University Hospital enrolled between 1 June 1996 and the 30 June 2012, and at Center of Thrombosis, Aalborg University Hospital enrolled between the 1 April 2008 and 31 December 2012. Each center recorded information on treatment indication, training, initiation of PSM, and INR measurements. Database parameters and patient training have previously been described in detail^13,14^. In brief, patients were educated to monitor the INR and adjust the VKA dose accordingly to achieve a designated target INR range. We compared these patients to similar patients with AF treated with dabigatran etexilate (approved for AF August 2011), rivaroxaban (approved for AF February 2012) or apixaban (approved for AF August 2012).

We used the unique civil registration number assigned to all Danish citizens^15^ to link data from three nationwide registries to obtain a dataset on patient treatment and comorbidity. Data on exposure to anticoagulant therapy and concomitant medications was obtained from the Danish National Prescription Registry^16^, which holds purchase date, Anatomical Therapeutic Chemical classification codes, and package size for every redeemed prescription in Denmark since 1994. Data on patient comorbidities and outcomes was obtained from the Danish National Patient Register^17^, which contains admission/discharge dates and International Classification of Diseases diagnoses for >99% of somatic hospital admissions in Denmark since 1977. Finally, demographic information was obtained from the Danish Civil Registration System^15^, which contains information on sex, date of birth, vital status, and emigration status.

### Study design

The study was conducted as a propensity-weighted cohort study. To qualify for enrolment in the PSM programme, patients should have non-valvular AF and be experienced with OAC treatment. The time of PSM initiation was baseline for this group. A control cohort was extracted from the population of Danish patients initiating DOAC treatment with either dabigatran etexilate 110 mg or 150 mg BID, rivaroxaban 15 mg or 20 mg OD, or apixaban 2.5 mg or 5 mg BID, with prior hospital diagnoses for non-valvular AF. Time of DOAC initiation was considered as baseline and was required to be within the period 1 January 2012 and 30 June 2014, thus avoiding inclusion of control patients initiated early after approval of dabigatran, when selective prescribing may have occurred.

As PSM patients are experienced VKA patients at enrolment, we also required that DOAC patients were switched from VKA with at least one VKA prescription within one year prior to initiating treatment with DOAC. Patients with prior hospital discharge codes for mitral stenosis or valvular replacement surgery indicating valvular AF were excluded. Previous studies have indicated that PSM patients represent a patient cohort with a relatively low comorbidity^11,12^. To reduce the risk of selection bias, we therefore restricted the control population to patients without comorbidity known to be associated with higher mortality, such as heart failure, cancer, chronic pulmonary disease or a Charlson index >2.

To control for possible confounding, we applied an inverse probability of treatment weighted (IPTW) analysis with weights defined to estimate the average treatment effect of the treated with focus on the PSM cohort^18,19^. The weights were based on the propensity score for self-managed OAC estimated using logistic regression. Predefined indicators of comorbidity and medication were included as predictors in the propensity model, and further risk strata were defined by the combination of sex, age (18–65, 65–75, >75 years of age) and OAC experience as duration (0–0.5, 0.5–1, 1–5, >5 years) between the first prescription of VKA and baseline. Indicators of disease burden were: CHA_2_DS_2_-Vasc and Charlson scores, prior bleeding, diabetes, peripheral artery disease, myocardial infarction, renal impairment, prescription redeemed within one year of acetylsalicylic acid, nonsteroidal anti-inflammatory drugs, treatment for hypertension, statins, amiodarone, proton pump inhibitors, digoxin or ACE-ARB inhibitor. We also required that each risk stratum should include at least one PSM patient and at least five eligible controls.

The study was approved by the Danish Data Protection Agency (ref. 2015-57-0001). Ethical approval and individual consent are not required for registry-based studies in Denmark. Data from the national registries was provided by Statistics Denmark.

### Outcome measures and comorbidity

The clinical outcome measures were ischaemic stroke (including systemic embolism), major bleeding (intracranial bleeding, gastrointestinal bleeding, various major bleedings or traumatic intracranial bleeding), all-cause stroke (ischaemic stroke, systemic embolism or intracranial bleeding) and all-cause death. ICD codes are listed in supplementary Table 1. To assess for possible inclusion bias, we applied a falsification analysis by investigating the endpoint of fractures of any type (hip, arms, or legs) as well as urinary tract infections: It was expected that these events were neither associated with the drugs investigated, neither with a time trend during the observation period (1998–2015). All hospital discharge data for endpoints were required to be primary or secondary diagnoses, non-ambulatory and non-emergency room codes. A follow-up of 2.5 years was applied for the analyses.

Baseline comorbidity was described according to medication and/or history of hospital discharge data. A prescription within one year before date of inclusion was used as indication of treatment. Both primary and secondary hospital discharge data back to 1994 was used for baseline comorbidity, when the ICD revision 10 was introduced. Briefly, comorbidity information consisted of cardiovascular and metabolic diseases, and we also collected data to establish the CHA_2_DS_2_-Vasc stroke risk score, the HAS-BLED bleeding risk score as well as Charlson’s score for overall comorbidity (supplementary Tables 1 and 2).

### Statistics

Patient characteristics at baseline are presented as proportions for discrete variables and means (SD) for continuous variables. The feasibility of obtaining unbiased estimates through the IPTW analysis by aligning baseline characteristics was evaluated by computing standardised differences in both the unweighted and weighted samples with standardised differences below 0.1 considered acceptable^20^. Person-time for DOAC patients and PSM patients were counted from initiation of treatment with DOAC and PSM, respectively, until the event of interest or 31 December 2015, emigration or death, whichever came first. Clinical endpoints are described by weighted incidence event rates calculated as number of events divided by weighted person-time at risk and by plotting Kaplan-Meier curves of mortality, and the cumulative incidence function (Aalen-Johansen) by time at risk for stroke and bleeding assuming death as a competing risk. Event rates among PSM patients were compared with the DOAC group and reported as hazard ratios (HR) estimated by weighted Cox proportional hazards models stratified according to the risk strata (defined above).

Patients with renal impairment and increased risk of bleeding are recommended to use low dose DOAC, and this may reflect a selected patient group with comorbidity not available in the registries^21^. We therefore repeated the analyses restricted to patients on standard dose DOAC. Furthermore, to ascertain the robustness of our analytic approach, we repeated the analysis as a 1:2 propensity nearest neighbour matched analysis and as a classical adjusted analysis, both using the coarsened exact match strata established above^22^.

Effect estimates were reported with 95% confidence intervals, and p-values < 0.05 were considered statistically significant. Analyses were performed using STATA version 14 (StataCorp LP, TX, USA). Author FS had full access to all data in the study and takes responsibility for its integrity and the data analysis.

## Results

The study population comprised 2,671 patients in the DOAC group and 534 in the PSM group. The proportion with full 2.5 years potential follow-up among DOAC users was 58%, whereas by design all PSM patients had full potential follow-up. In the DOAC group, 68% of patients started treatment with dabigatran, 23% with rivaroxaban and 9% with apixaban. Within 2.5 years, 20% of the DOAC patients redeemed a prescription for warfarin at least once. In the PSM group, the number of patients switching to DOAC was low, mainly as the end of 2.5 years of follow-up was reached before introduction of DOACs for AF.

**Figure 1:**
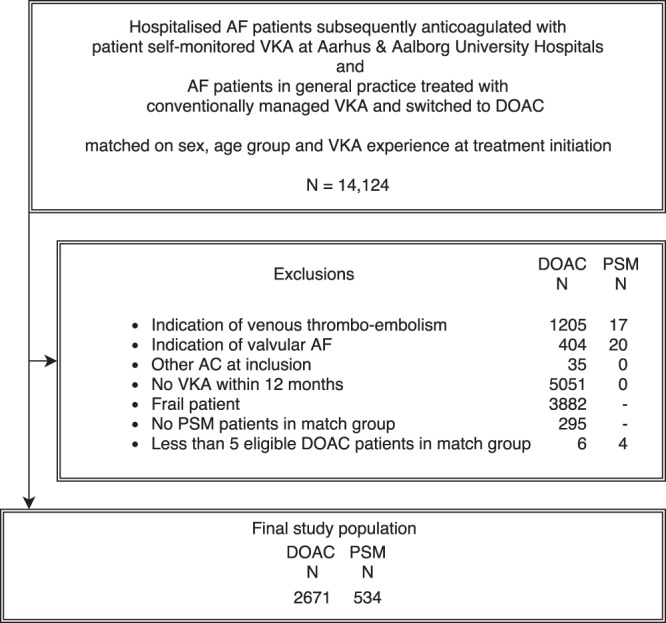
Flowchart of patient inclusion. AC: anticoagulant; AF: atrial fibrillation; DOAC: direct oral anticoagulant; PSM: Patient self-management group; VKA: vitamin-K antagonist.

During the inclusion period, a total of 575 self-managed patients with AF were identified at Aarhus University Hospital and Aalborg University Hospital of which 37 had additional indications for OAC, such as VTE or valvular AF, and were thus excluded. A total of 13,549 patients with AF initiated a DOAC in the inclusion period. Among these, we excluded patients due to prior VTE (N = 1,205), valvular AF (N = 404), more than one OAC type initiated (N = 35) or no prior warfarin use (N = 5,051). In the DOAC population, a total of 3,882 patients were identified with diagnostic information related to diseases with an expected poor prognosis (heart failure, cancer, chronic pulmonary disease) or high comorbidity as indicated by a Charlson score >2. Eventually, we removed risk strata with no cases or less than five eligible controls (N = 295 of which four were cases, Fig. 1).

**Figure 2:**
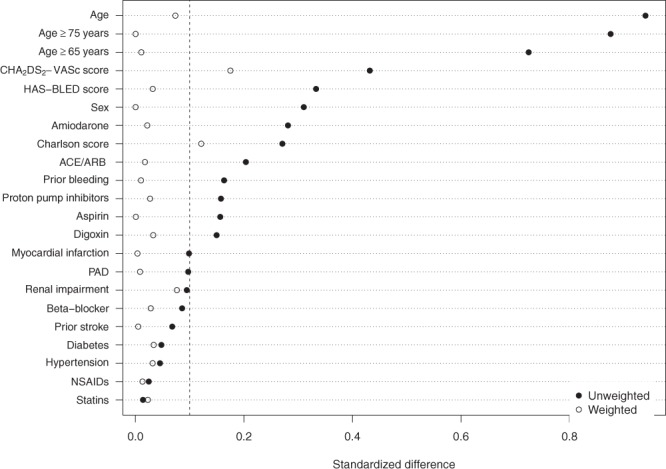
Standardised baseline differences before and after IPTW analysis. ACE/ARB, angiotensin converting enzyme inhibitors/angiotensin II receptor blockers IPTW, inverse probability of treatment weighted, NSAIDs, nonsteroidal anti-inflammatory drugs PAD, peripheral artery disease.

Unweighted baseline characteristics and demographics of the study population are shown in Table 1. The selected DOAC population was older and with a higher proportion of patients with prior bleeding, slightly more with hypertension and use of proton pump inhibitors. The PSM population included 20.4% with indication of heart failure, and patients with cancer (all types including any previous cancers) or chronic pulmonary disease accounted for 9.7% and 2.2%, respectively. By design, these patients were excluded in the DOAC cohort.. Standardised differences in the unweighted and the IPTW population are shown in Fig. 2. Among >20 standardised differences, two weighted parameters remained slightly above 0.1: the CHA_2_DS_2_-Vasc stroke risk score and Charlson’s comorbidity score for overall comorbidity. As detailed in Table 1, the CHA_2_DS_2_-Vasc score was slightly higher among patients treated with PSM, whereas Charlson score was slightly lower. Overall, Fig. 2 indicate that the differences between the treatment groups are efficiently reduced in the IPTW population.

**Table 1:** Baseline characteristics for DOAC and self-management groups.

Variable	Self-management group	DOAC group unweighted	DOAC group ATT weighted
Number of patients	534	2671	2671
2.5 years potential follow-up, % (N)	100 (534)	57.7 (1542)	61.9 (328)
Female sex, % (N)^§^	23.6 (126)	37.7 (1008)	23.6 (126)
Age, mean (SD)^§^	63.1 (7.4)	71.6 (10.3)	63.7 (8.2)
Age ≥ 65years, % (N)	43.1 (230)	76.5 (2043)	42.5 (227)
Age ≥ 75years, % (N)	4.1 (22)	36.4 (971)	4.1 (22)
Duration of prior VKA therapy, months, mean (SD)^§^	32.5 (34.1)	41.8 (46.8)	38.3 (42.8)
CHA_2_DS_2_-Vasc stroke risk score, mean (SD)	2.2 (1.4)	2.8 (1.5)	1.9 (1.4)
HAS-BLED bleeding score, mean (SD)	1.9 (1.1)	2.3 (1.1)	1.9 (1.2)
Charlson score, mean (SD)	0.8 (1.1)	0.5 (0.7)	0.6 (0.8)
Prior stroke, % (N)	18.2 (97)	20.7 (629)	18.4 (98)
Prior bleeding, % (N)	11.8 (63)	17.6 (469)	11.5 (61)
Hypertension, % (N)	62.9 (336)	60.7 (1622)	61.4 (327)
Diabetes, % (N)	10.9 (58)	12.4 (331)	9.8 (52)
Myocardial infarction, % (N)	9.0 (48)	6.4 (170)	8.9 (47)
Peripheral artery disease, % (N)	3.4 (18)	5.4 (143)	3.2 (17)
Moderate/severe kidney impairment, % (N)	2.6 (14)	1.3 (35)	4.0 (21)
Heart failure, % (N)	20.4 (109)	0.0 (0)^⤈^	0.0 (0)^⤈^
Cancer, % (N)	9.7 (52)	0.0 (0)^⤈^	0.0 (0)^⤈^
Chronic obstructive pulmonary disease, % (N)	2.2 (12)	0.0 (0)^⤈^	0.0 (0)^⤈^
Aspirin, % (N)	36.5 (195)	29.2 (708)	36.6 (195)
NSAIDs, % (N)	20.2 (108)	19.2 (514)	19.7 (105)
Statins, % (N)	45.7 (244)	46.4 (1239)	44.6 (237)
Beta-blocker, % (N)	73.4 (392)	69.5 (1875)	72.2 (384)
Proton pump inhibitor, % (N)	15.2 (81)	21.2 (567)	16.2 (86)
Amiodarone, % (N)	14.8 (79)	6.3 (167)	14.0 (75)
Digoxin, % (N)	30.5 (163)	23.9 (638)	29.0 (155)
ACE or ARB inhibitors, % (N)	54.3 (290)	44.2 (1180)	53.4 (285)

^§^Variables used for coarsened exact match. ^⤈^Excluded by design.

ACE/ARB: Angiotensin converting enzyme inhibitors, angiotensin II receptor blockers; ATT: Average treatment effect of the treated; DOAC: Direct oral anticoagulant; SD: Standard deviation; NSAID: Nonsteroidal anti-inflammatory drugs; CHA_2_DS_2_-Vasc: stroke risk score; HAS-BLED: bleeding risk score.

**Figure 3:**
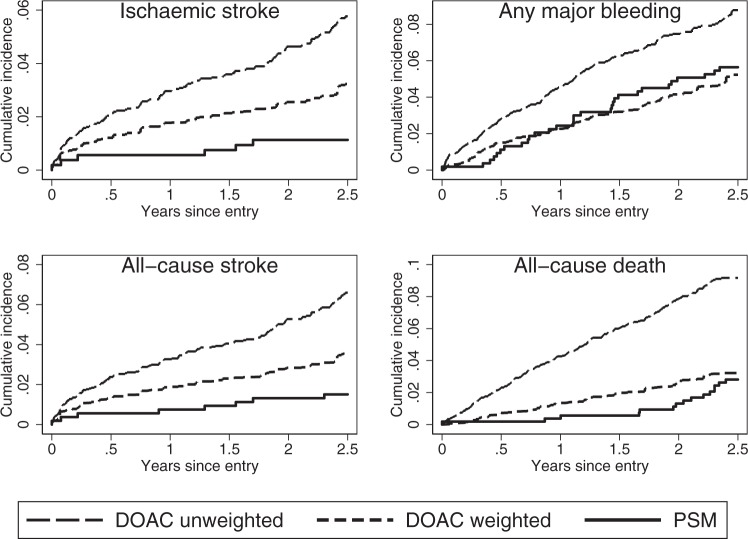
Cumulative incidence functions for ischaemic stroke, bleeding and death. Ischaemic stroke includes systemic embolism. DOAC: direct oral anticoagulant; PSM: Patient self-management group.

Risk profiles in terms of cumulative incidence are presented in Fig. 3, highlighting the low incidence of ischaemic stroke among PSM patients as only six events were observed. The risk of a major bleeding event approached 6% at follow-up after 2.5 years for both PSM and the comparable (weighted) DOAC population. Very few deaths were observed among the PSM patients during the first 1.5 years of PSM treatment; after 2.5 years, mortality approached 4%, which was slightly below the comparable DOAC population.

At follow-up after 2.5 years, ischaemic stroke was observed with a rate of 0.5 per 100 person years among PSM patients (Table 2). This was significantly lower than in the DOAC group (HR 0.27 (95% CI 0.11–0.68). Major bleeding was observed at a weighted rate of about 2.3 in both groups, thus comparable with a HR of 1.06 (95% CI 0.69–1.63). The combined ischaemic and hemorrhagic all-cause stroke outcome was lower in PSM group with weighted rates 0.61 vs 1.45 in the DOAC group (HR 0.36 95% CI 0.16–0.78). The analysis of all-cause death showed a rate of 1.1 per 100 person years among PSM patients, whereas the rate in the DOAC group was 1.3, corresponding to a HR of 0.67 (95% CI 0.39–1.17). A landmark analysis obtained by restricting the survival analysis to patients alive one year after inclusion resulted in a HR of 1.09 (95% CI 0.57–2.09).

**Table 2:** Event rates and hazard ratios for endpoints in DOAC and PSM cohorts after 2.5 years of follow-up.

	Events N	Crude event rate (%/year)	IPTW ATT event rate (%/year)	IPTW ATT hazard ratio (HR) (95% CI)
**Ischaemic stroke incl SE**				
DOAC	134	2.36	1.30	Reference
PSM	6	0.46	0.46	0.27 (0.11–0.68)
**Major bleeding**				
DOAC	211	3.79	2.13	Reference
PSM	30	2.32	2.32	1.06 (0.69–1.63)
**All-cause stroke**				
DOAC	153	2.71	1.45	Reference
PSM	8	0.61	0.61	0.36 (0.16–0.78)
**All-cause death**				
DOAC	228	3.92	1.32	Reference
PSM	15	1.13	1.13	0.67 (0.39–1.17)
**Fractures (control outcome)**				
DOAC	174	3.09	1.78	Reference
PSM	18	1.37	1.37	0.78 (0.45–1.36)
**Urinary tract infection (control outcome)**				
DOAC	114	2.00	0.71	Reference
PSM	7	0.53	0.53	0.64 (0.27–1.52)
**Landmark analysis, All-cause death after one year in study**				
DOAC	115	3.92	1.0	Reference
PSM	14	1.77	1.77	1.09 (0.57–2.09)

ATT = average treatment on the treated (self-management group); CI: Confidence Interval; DOAC: direct oral anticoagulant; HR: Hazard Ratio; IPTW = Inverse probability of treatment weighting; PSM: Patient self-management group; SE: systemic embolism.

The falsification analysis of the two control outcomes, fractures and urinary tract infection, displayed lower rates among the PSM patients, but both with unity within the confidence intervals of the HRs.

During the 2.5 years of follow-up, a total of 23 and only two intracranial bleedings were observed in the DOAC and PSM groups, respectively. In the weighted cohorts, these numbers corresponded to rates of 0.19 and 0.15. Due to low number of events, we did not further explore this difference.

Table 3 shows hazard ratios for endpoints contrasting DOAC vs PSM cohorts after additional sensitivity analyses with findings similar to the ones summarised above. Accordingly, treatment with PSM compared to DOAC was associated with a significantly lower risk of both all-cause and ischaemic stroke, whereas no difference was observed for major bleeding. The risk of death was numerically lower in the PSM group.

**Table 3:** Hazard ratios for endpoints in DOAC and PSM cohorts after sensitivity analyses and 2.5 years follow-up.

	Main analysis IPTW ATT hazard ratio (HR) (95% CI)	Propensity matched 1:2 hazard ratio (H) (95% CI)	Standard dose IPTW ATT hazard ratio (HR) (95% CI)	Adjusted hazard ratio (HR) (95% CI)
**Ischaemic stroke incl SE**				
DOAC	Reference	Reference	Reference	Reference
PSM	0.27 (0.11–0.68)	0.28 (0.12–0.67)	0.28 (0.10–0.77)	0.26 (0.11–0.57)
**Major bleeding**				
DOAC	Reference	Reference	Reference	Reference
PSM	1.06 (0.69–1.63)	0.89 (0.57–1.38)	0.97 (0.57–1.64)	1.00 (0.70–1.44)
**All-cause stroke**				
DOAC	Reference	Reference	Reference	Reference
PSM	0.36 (0.16–0.78)	0.34 (0.16–0.72)	0.38 (0.16–0.91)	0.33 (0.16–0.65)
**All-cause death**				
DOAC	Reference	Reference	Reference	Reference
PSM	0.67 (0.39–1.17)	0.71 (0.39–1.31)	0.83 (0.38–1.85)	0.76 (0.46–1.26)
**Fractures (control outcome)**				
DOAC	Reference	Reference	Reference	Reference
PSM	0.78 (0.45–1.36)	0.81 (0.46–1.41)	0.76 (0.40–1.843)	0.76 (0.46–1.21)
**Urinary tract infection (control outcome)**				
DOAC	Reference	Reference	Reference	Reference
PSM	0.64 (0.27–1.52)	0.43 (0.19–0.99)	0.58 (0.20–1.68)	0.66 (0.31–1.39)
**Landmark analysis, All-cause death after one year in study**				
DOAC	Reference	Reference	Reference	Reference
PSM	1.09 (0.57–2.09)	1.06 (0.53–2.10)	1.34 (0.51–3.50)	1.23 (0.72–2.09)

ATT = average treatment on the treated (self-management group); CI: Confidence Interval; DOAC: direct oral anticoagulant; HR: Hazard Ratio; IPTW = Inverse probability of treatment weighting; PSM: Patient self-management group; SE: systemic embolism.

Increasing the proportion of DOAC patients with full 2.5 years potential follow-up by using an earlier date for stop of inclusion did not alter the results substantially. Neither did restriction to patients treated with standard dose DOAC. Altering the analytical approach by using 1:2 direct matching or traditional adjusted analyses resulted in comparable rates and HRs.

## Discussion

This is the largest cohort study evaluating self-managed treatment with VKA in patients with AF. Using propensity weights to account for differences in comorbidity, we compared the effectiveness and safety of DOAC versus self-managed anticoagulant treatment with VKA in patients with AF and have shown that PSM of properly trained patients is safe and effective in a real-life setting. We found that a strategy of PSM was associated with a significantly lower risk of both ischaemic and all-cause stroke compared to treatment with DOAC, whereas no significant differences were observed for major bleeding and mortality. These novel findings indicate that self-managed OAC with VKA should be considered more frequently as an anticoagulant strategy for patients with AF. When interpreting these results it should be acknowledged that falsification analyses indicated a risk of some residual confounding. Moreover, not all patients with AF are eligible for self-managed oral anticoagulation^23^.

Oral anticoagulant treatment remains the preferred strategy to reduce the risk of stroke in patients with AF, and international guidelines recommend initiation of DOAC instead of VKA in patients who are eligible for both^7,8^. Two classes of DOAC are available: direct thrombin inhibitors (dabigatran) and factor Xa inhibitors (e.g. rivaroxaban and apixaban). All these drugs have shown at least non-inferiority to conventionally managed VKA treatment^24^, and in some cases superior efficacy for the primary endpoint of ‘stroke and systemic embolism’ (dabigatran 150 mg twice daily + apixaban)^3,4^. In our study, PSM was associated with a significantly lower risk of all-cause stroke and systemic embolism and also a lower risk of ischaemic stroke.

The safety and efficacy of self-managed treatment with VKA has been the subject of several clinical trials. A meta-analysis of individual patient data from 11 trials showed that PSM is a safe option for suitable patients with a significant reduction in thromboembolic events compared to conventionally managed VKA^9^. In 2017, a comprehensive systematic review of 28 studies concluded that compared to conventionally managed VKA, PSM was associated with a significant reduction in thromboembolic events (RR 0.58; 95% CI 0.45–0.74), with no significant effects on bleeding or all-cause mortality^10^. The findings of our study are consistent with this conclusion; however, in the systematic review only 2 studies included patients with AF, thus stressing the need for more data on PSM in patients with AF.

Even more important is the fact that there is a clear need for studies comparing PSM with DOAC, since both anticoagulant strategies have proved superior to conventionally managed VKA^3–6,9^. Contrary to the solid evidence for treatment with either PSM or DOAC, no studies have directly compared these treatment strategies in patients with AF. A previous network meta-analysis concluded that the efficacy of PSM is comparable to VKA standard care and DOAC, but this analysis only included 203 patients with AF^25^. Similarly, an indirect comparison of dabigatran with home-monitoring (self-testing + PSM) suggested that these anticoagulant strategies have similar impact on thrombosis, bleeding and mortality, although with confidence in the estimate of effect being ‘low to very low’^26^. Our study adds to the growing evidence supporting PSM as an important option in patients with AF.

Intracranial bleeding is the most feared and devastating complication of anticoagulant treatment, because it carries a very high risk of morbidity and mortality^27^. Importantly, treatment with DOAC in phase III trials of patients with AF was associated with a significantly lower risk of intracranial bleeding, regardless of the type and dose of DOAC tested^28^. In our study, the risk of intracranial bleeding was low in both groups, but numerically lower in the PSM group. It is well-known that the risks of VKA-associated intracranial bleeding are proportional to the intensity of anticoagulation^29^, and the low risk of intracranial bleeding in PSM patients is at least partly explained by the high quality of anticoagulant control with INR values within the target range in >70% of patients^14^.

The reason for the similar rate of major bleeding events during PSM and DOAC is likely multifaceted. Although PSM is a high quality VKA treatment in terms of time in therapeutic range^14^, it is known that the majority of bleeding events occur within therapeutic range^30^. Furthermore, the rigorous monitoring of PSM patients likely results in improved adherence compared to patients receiving usual care with VKA or DOAC^31^. Finally, when interpreting the absolute risk of bleeding, it should be acknowledged that this is likely to be lower in patients included after several years of anticoagulant treatment.

Within recent years, there has been a large increase in the use of DOAC for thromboprophylaxis in patients with AF^32,33^. This is consistent with international guidelines^7,8^, and is explained by the favourable results in phase III trials with at least similar efficacy as VKA and a lower risk of major bleeding, especially intracranial bleeding^3–6^. Also, there are fewer drug interactions than for VKA, and the predictable pharmacology eliminates the need for laboratory monitoring of the anticoagulant effect and frequent dose adjustments, and DOAC thus provides a safe and convenient alternative to conventionally managed treatment with VKA. One may therefore question if PSM with point-of-care devices is still a viable treatment option. However, not all patients are eligible for DOAC, if e.g. patients have severe renal failure, mechanical heart valves or a considerably increased risk of gastrointestinal bleeding. Importantly, one should also consider PSM to some patients eligible for treatment with DOAC. PSM seems to be cost-effective^34^ and, compared to conventional VKA treatment, it is associated with a higher patient satisfaction with OAC and quality of life^35,36^.

### Strengths and limitations

The two-center inclusion and the long-term follow-up are important strengths, and a high external validity of our data is likely. All AF patients referred for PSM in the study period were included. Our comparator group was sampled from the entire Danish population and had complete follow-up through nationwide registries, thus avoiding the selection bias traditionally hampering clinic-based studies. Baseline characteristics of the unmatched cohorts of PSM and DOAC users were largely comparable, and did thus not indicate major differences in prescribing patterns. Selection of patients for PSM is dependent on the referral pattern, however this selection bias is limited because the cost of PSM in Denmark is reimbursed for all patients. Limitations of our study mainly relate to its observational nature and the relatively low number of patients, which e.g. did not allow for comparison of effectiveness and safety across different DOACs. Although we applied propensity weighting to account for baseline differences and differential prescribing behavior, some residual and unmeasured confounding may persist as indicated by the falsification endpoint analyses. Our estimates of effectiveness and safety are based on propensity-weighting conducted in a setting with free access to health services, thus largely eliminating e.g. referral bias. We had no access to laboratory, anthropometric, or socioeconomic data. We also lacked data on lifestyle factors such as alcohol consumption and smoking that may influence outcomes in patients with AF. However, we were able to control for hospital diagnoses of alcohol-related conditions and many other lifestyle-related diseases including diabetes, cardiovascular disease, and chronic pulmonary diseases, which to some extent serve as markers of the above-mentioned lifestyle factors. Finally, sensitivity analyses did not change conclusions, suggesting high internal validity.

## Conclusions

Self-managed OAC was associated with a lower risk of ischaemic and all-cause stroke compared to treatment with DOAC, whereas no significant differences were observed for major bleeding and mortality. This study shows that in properly trained patients, self-managed OAC could be an effective and safe alternative to DOAC as long-term anticoagulant treatment for stroke prevention in patients with AF. These findings should be confirmed in a randomised controlled trial.

## Electronic supplementary material

Supplementary tables

## Data Availability

The datasets analysed for this study are available from the corresponding author upon reasonable request.
