# Supplementary material for: Effectiveness and safety of self-managed oral anticoagulant therapy compared with direct oral anticoagulants in patients with atrial fibrillation
Source: Sci Rep. 2018 Oct 25;8:15805. doi: 10.1038/s41598-018-33531-7 (PMC6202319; doi:10.1038/s41598-018-33531-7)
Supplement: Supplementary file 1 — Supplementary tables [file 41598_2018_33531_MOESM1_ESM.docx]

*Supplementary tables to the manuscript:*

**Effectiveness and safety of self-managed oral anticoagulant therapy compared with direct oral anticoagulants in patients with atrial fibrillation**

Erik Lerkevang Grove, MD, PhD^1,2^

Flemming Skjøth, MSc, PhD^3,4^

Peter Brønnum Nielsen, MSc, PhD^3,6^

Thomas Decker Christensen, MD, PhD^2,5^

Torben Bjerregaard Larsen, MD, PhD^3,6*^

^1^Department of Cardiology, Aarhus University Hospital, Aarhus, Denmark

^2^Department of Clinical Medicine, Faculty of Health, Aarhus University, Denmark

^3^Aalborg Thrombosis Research Unit, Department of Clinical Medicine, Faculty of Health, Aalborg University, Aalborg, Denmark

^4^Unit of Clinical Biostatistics, Aalborg University Hospital, Aalborg, Denmark

^5^Department of Cardiothoracic and Vascular Surgery, Aarhus University Hospital, Aarhus, Denmark

^6^Department of Cardiology, Aalborg University Hospital, Aalborg, Denmark

**Supplementary Table 1. ICD-10/ATC codes used to identify endpoints, comorbidity and comedication**

|  | **International Classification of Diseases 10th revision (ICD-10) code** | **Anatomical Therapeutic Chemical (ATC) code** |
| --- | --- | --- |
| **Condition** |  |  |
| Congestive heart failure^*^ | I11.0; I13.0; I13.2; I42.0; I50 | and C03C |
| Left ventricular dysfunction^*^ | I50.1; I50.9 |  |
| Hypertension^*§^ |  | See specified definition^a^ |
| Diabetes mellitus^*^ | E10.0; E10.1; E10.9; E11.0; E11.1; E11.9 | or A10 |
| Ischaemic stroke^*§^ | I63; I64.9 |  |
| Systemic embolism^*^ | I74 |  |
| Transient ischaemic attack^*§^ | G45 |  |
| Pulmonary embolism | I26; O882 |  |
| Peripheral vascular disease^*^ | I702; I703; I704; I705; I706; I707; I708; I709; I71; I739 |  |
| Myocardial infarction^*^ | I21; I23 |  |
| Deep venous thromboembolism | I801-I803; I808; I809; I81; I822; I823; I828; I829; O223; O229; O225; O871; O879; O873 |  |
| Abnormal renal function^§^ | I12; I13; N00-N05; N07; N11; N14; N17-N19; Q61 |  |
| Abnormal hepatic function^§^ | B15.0; B16.0; B16.2; B19.0; K70.4; K72; K76.6; I85 |  |
| Intracranial bleeding^§^ | I60-I62 |  |
| Gastrointestinal bleeding^§^ | K250; K252; K254; K260; K262; K264; K270; K272; K274; K280; K282; K290; K920; K921; K922 |  |
| Traumatic intracranial bleeding^§^ | S06.3C; S06.4; S06.5; S06.6 |  |
| Various major bleedings^§^ | D62; J94.2; H11.3; H35.6; H43.1; N02; N95; R04; R31; R58 |  |
| Anemia | D5; D60; D61; D62; D63; D64 |  |
| Alcohol intake^§^ | E22.4; E52.9A; F10; G31.2; G62.1; G72.1; I42.6; K29.2; K70; K86.0; L27.8A; O35.4M; T51; Z71.4; Z72.1 |  |
| Atrial fibrillation | I48 |  |
| Valvular AF | I05; Z952; Z953; Z954 |  |
| Cancer | C |  |
| Chronic obstructive pulmonary disease | J44 |  |
| Moderate/severe renal disease | I12; I13; N00; N01; N02; N03; N04; N05; N07; N11; N14; N17; N18; N19; Q61 |  |
| Knee or hip replacement | SKS: NGB; NGC; NGU; NFB; NFC; NFU; |  |
| Any major surgery | SKS: A; B; D; F; G; H; I; J; K; M; N; P; Y |  |
| **Medication** |  |  |
| Amiodarone |  | C01BD01 |
| Apixaban |  | B01AF02 |
| Aspirin |  | B01AC06 |
| Beta-blocker |  | C07 |
| Dabigatran |  | B01AE07 |
| Digoxin |  | C01AA05 |
| Non-steroidal  anti-inflammatory drugs |  | M01A |
| Phenprocoumon |  | B01AA04 |
| Proton-pump inhibitors |  | A02BC |
| Renin-angiotensin inhibitor (ARB or ACE inhibitor) |  | C09 |
| Rivaroxaban |  | B01AF01 |
| Statins |  | C10 |
| Warfarin |  | B01AA03 |

ACE, angiotensin-converting enzyme; ARB, angiotensin receptor blocker.

^*^ Included in CHA_2_DS_2_VASc score. ^§^ Included in HAS-BLED score.

^a^We identified subjects with hypertension from combination treatment with at least two of the following classes of antihypertensive drugs:

I. Alpha adrenergic blockers (C02A, C02B, C02C)

II. Non-loop diuretics (C02DA, C02L, C03A, C03B, C03D, C03E, C03X, C07C, C07D, C08G, C09BA, C09DA, C09XA52)

III. Vasodilators (C02DB, C02DD, C02DG, C04, C05)

IV. Beta blockers (C07)

V. Calcium channel blockers (C07F, C08, C09BB, C09DB)

VI. Renin-angiotensin system inhibitors (C09).

**Supplementary Table 2. Definition of CHA_2_DS_2_VASc and HAS-BLED risk scores.**

| **Risk score** | **Points** |
| --- | --- |
| CHA_2_DS_2_VASc^a^ |  |
| Congestive heart failure or left ventricular dysfunction | 1 |
| Hypertension | 1 |
| Age ≥ 65 years | 1 |
| Age ≥ 75 years | 1 |
| Diabetes mellitus | 1 |
| Stroke (ischaemic stroke, transient ischaemic disease or systemic embolism) | 2 |
| Vascular disease (myocardial infarction, peripheral arterial disease, or aortic plaque) | 1 |
| Sex category (female) | 1 |
| HAS-BLED^b^ |  |
| Hypertension | 1 |
| Abnormal renal function | 1 |
| Abnormal hepatic function | 1 |
| Stroke (ischaemic stroke or transient ischaemic attack) | 1 |
| Bleeding | 1 |
| Labile international normalized ratio^c^ | 1 |
| Elderly age (≥ 65 years) | 1 |
| Drugs (aspirin, clopidogrel, or non-steroidal anti-inflammatory drugs) | 1 |
| Alcohol intake | 1 |

^a^Reflects stroke risk in atrial fibrillation patients not in anticoagulant therapy ^1^.

^b^Reflects bleeding risk in atrial fibrillation patients undergoing anticoagulant therapy ^2^.

^c^Not included due to unavailable information

**Supplementary Table 3. Definition of Charlson´s comorbidity index ^3^**

| **Weight** | **Condition** | **ICD-10** | **ICD-8** |
| --- | --- | --- | --- |
| 1 | Myocardial infarction | I21, I22, I23 | 410 |
| 1 | Congestive heart failure | I50, I110, I130, I132 | 42709, 42710, 42711, 42719, 42899, 78249 |
| 1 | Peripheral vascular disease | I70, I71, I72, I73, I74, I77 | 440, 441, 442, 443, 444, 445 |
| 1 | Cerebrovascular disease | I60, I61, I62, I63, I64, I65, I66, I67, I68, I69, G45, G46 | 430, 431, 432, 433, 434, 435, 436, 437, 438 |
| 1 | Dementia | F00, F01, F02, F03, F051, G30 | 29009, 29010, 29011, 29012, 29013, 29014, 29015, 29016, 29017, 29018, 29019, 29309 |
| 1 | Cronic pulmonary disease | J40, J41, J42, J43, J44, J45, J46, J47, J60, J61, J62, J63, J64, J65, J66, J67 J684, J701, J703, J841, J920, J961, J982, J983 | 490, 491, 492, 493, 515, 516, 517, 518 |
| 1 | Connective tissue disease | M05, M06, M08, M09, M30, M31, M32, M33, M34, M35, M36, D86 | 712, 716, 734, 446, 13599 |
| 1 | Ulcer disease | K221, K25, K26, K27, K28, | 53091, 53098, 531, 532, 533, 534 |
| 1 | Mild lever disease | B18, K700, K701, K702, K703, K709, K71, K73, K74, K760 | 571, 57301, 57304 |
| 1 | Diabetes Mellitus | E10, E11, E12, E13, E14, O240, O241, O242, O243, O245, O246, O247, O248, O249, H360 | 249, 250 |
| 2 | Hemiplegia | G81, G82 | 344 |
| 2 | Moderate to severe renal disease | I12, I13, N00, N01, N02, N03, N04, N05, N07, N11, N14, N17, N18, N19, Q61 | 403, 404, 580, 581, 582, 583, 584, 59009, 59319, 7531, 792 |
| 2 | Diabetes with chronic complications | E102, E103, E104, E105, E106, E107, E108, E112, E113, E114, E115, E116, E117, E118 | 24901, 24902, 24903, 24904, 24905, 24908, 25001, 25002, 25003, 25004, 25005, 25008 |
| 2 | Any tumor | C0-6, C70, C71, C72, C73, C74, C75 | 14-18, 190, 191, 192, 193, 194 |
| 2 | Leukemia | C91-95 | 204-7 |
| 2 | Lymphoma | C81-85, C88, C90, C96 | 200-203, 27559 |
| 3 | Moderate to severe liver disease | B150, B160, B162, B190, K704, K72, K766, I85 | 07000, 07002, 07004, 07006, 07008, 57300, 4560 |
| 6 | Metastatic solid tumor | C76-80 | 195-199 |
| 6 | AIDS | B21 B22 B23 B24 | 07983 |

**References**

1. Lip, G.Y., Nieuwlaat, R., Pisters, R., Lane, D.A. & Crijns, H.J. Refining clinical risk stratification for predicting stroke and thromboembolism in atrial fibrillation using a novel risk factor-based approach: the euro heart survey on atrial fibrillation. *Chest* **137**, 263-272 (2010).

2. Pisters, R.*, et al.* A novel user-friendly score (HAS-BLED) to assess 1-year risk of major bleeding in patients with atrial fibrillation: the Euro Heart Survey. *Chest* **138**, 1093-1100 (2010).

3. Charlson, M.E., Pompei, P., Ales, K.L. & MacKenzie, C.R. A new method of classifying prognostic comorbidity in longitudinal studies: development and validation. *Journal of chronic diseases* **40**, 373-383 (1987).
